# The Link Between Problematic Internet Use, Physical Activity, and Mental Health: Implications for Depression and Anxiety

**DOI:** 10.1192/j.eurpsy.2025.1641

**Published:** 2025-08-26

**Authors:** M. Theodoratou, I. Patiri, D. Katsarou, E. Efthymiou, M. Sofologi, G. A. Kougioumtzis, S. Papadopoulou

**Affiliations:** 1 Social Sciences; 2 Hellenic Open University, Patras; 3 Psychology, University of Aegean, Aegean, Greece; 4 Psychology, University of Zayed, Emirates, United Arab Emirates; 5 Psychology, University of Ioannina, Ioannina; 6 Turkish studies, National Kapodistrian University of Athens, Athens; 7 Speech Therapy, University of Ioannina, Ioannina, Greece

## Abstract

**Introduction:**

The Internet has become an indispensable component of contemporary life, enabling communication, work, and leisure activities. However, with its increasing use, concerns are emerging regarding problematic internet use (PIU) and its effects on physical and mental health. PIU has been associated with a range of adverse outcomes, including reduced physical activity, depression, and anxiety. It is of paramount importance to gain insight into the complex relationship between internet use, physical activity, and mental health in order to develop effective interventions that can mitigate the adverse effects associated with these factors.

**Objectives:**

The objective was to quantify the extent of internet usage among participants and to analyze its potential association with physical activity, depression, and anxiety.

**Methods:**

The study sample consisted of 119 individuals from Patras. The data were collected using a set of validated questionnaires, including:

The Problematic Internet Use Questionnaire was employed to ascertain the extent of problematic internet use (PIU).

The International Physical Activity Questionnaire (IPAQ) was employed to assess the frequency and intensity of the participants’ physical activity.

The Patient Health Questionnaire (PHQ-9) was employed to evaluate the presence and severity of depressive symptoms.

The Generalized Anxiety Disorder (GAD-7) scale was employed to assess the levels of anxiety experienced by the participants.

**Results:**

The results indicate that a moderate level of problematic internet use is prevalent among the participants, with the majority spending more than four hours online daily. The analysis demonstrated that while increased internet use did not significantly impact the frequency or intensity of physical activity, there was a significant positive correlation between problematic internet use (PIU) and both depression and anxiety levels. As problematic internet use (PIU) increased, so did the symptoms of depression and anxiety.

**Conclusions:**

The study highlights the potential mental health risks associated with problematic internet use. Despite no observed impact on physical activity levels, the strong association between PIU and elevated depression and anxiety symptoms suggests a need for targeted interventions. Addressing PIU could play a crucial role in improving mental health outcomes, emphasizing the importance of developing strategies to manage internet usage effectively.

**Disclosure of Interest:**

None Declared

